# Extrahepatic cholangiocyte obstruction is mediated by decreased glutathione, Wnt and Notch signaling pathways in a toxic model of biliary atresia

**DOI:** 10.1038/s41598-020-64503-5

**Published:** 2020-05-05

**Authors:** Sophia Fried, Dafna Gilboa, Adi Har-Zahav, Pierre-Marie Lavrut, Yu Du, Sara Karjoo, Pierre Russo, Raanan Shamir, Rebecca G. Wells, Orith Waisbourd-Zinman

**Affiliations:** 1 Institute for Gastroenterology, Nutrition and Liver Diseases, Schneider Children’s Medical Center of Israel, Petach Tikva, Israel; 2 Sackler Faculty of Medicine, Tel-Aviv University, Tel-Aviv, Israel; 3 Department of Pathology, Hospices Civils de Lyon, Lyon, France; 4 Division of Gastroenterology, Department of Medicine, Perelman School of Medicine at the University of Pennsylvania, Philadelphia, PA United States; 5 Johns Hopkins School of Medicine, Baltimore, Maryland United States; 6 Department of Pathology and Laboratory Medicine, The Children’s Hospital of Philadelphia, Philadelphia, PA United States; 7 Division of Gastroenterology, Hepatology, and Nutrition, Department of Pediatrics, The Children’s Hospital of Philadelphia, Philadelphia, PA United States

**Keywords:** Bile ducts, Bile duct disease

## Abstract

Biliary atresia is a neonatal liver disease with extrahepatic bile duct obstruction and progressive liver fibrosis. The etiology and pathogenesis of the disease are unknown. We previously identified a plant toxin, biliatresone, responsible for biliary atresia in naturally-occurring animal models, that causes cholangiocyte destruction in *in-vitro* models. Decreases in reduced glutathione (GSH) mimic the effects of biliatresone, and agents that replenish cellular GSH ameliorate the effects of the toxin. The goals of this study were to define signaling pathways downstream of biliatresone that lead to cholangiocyte destruction and to determine their relationship to GSH. Using cholangiocyte culture and 3D cholangiocyte spheroid cultures, we found that biliatresone and decreases in GSH upregulated RhoU/Wrch1, a Wnt signaling family member, which then mediated an increase in Hey2 in the NOTCH signaling pathway, causing downregulation of the transcription factor Sox17. When these genes were up- or down-regulated, the biliatresone effect on spheroids was phenocopied, resulting in lumen obstruction. Biopsies of patients with biliary atresia demonstrated increased RhoU/Wrch1 and Hey2 expression in cholangiocytes. We present a novel pathway of cholangiocyte injury in a model of biliary atresia, which is relevant to human BA and may suggest potential future therapeutics.

## Introduction

Biliary Atresia (BA) is a pediatric liver disease which occurs in neonates and is characterized by extrahepatic bile duct obstruction and progressive liver fibrosis. Although rare (1:8,000-1:15,000 live births globally)^1^, BA is the main indication for pediatric liver transplant and a major cause of liver-related morbidity and mortality in the pediatric population^1–3^. The etiology of BA remains unknown. Several studies suggest that BA results from an environmental insult, such as virus or toxin exposure, while autoimmunity seems to play a significant role in disease progression. Genome-wide association studies have identified susceptibility genes potentially linked to BA, but it is not primarily a genetic disease^4–6^. Unlike other forms of biliary fibrosis, the initial injury in BA is in the extrahepatic bile ducts (EHBDs). There is growing evidence that the insult to the EHBD is prenatal^7–11^.

We previously identified a plant-derived biliary toxin, biliatresone, that likely causes BA in Australian livestock, and was found to cause selective extra-hepatic biliary obstruction in zebrafish larvae. In mouse cholangiocytes cultured in 3D as spheroids, biliatresone treatment resulted in loss of lumens, cholangiocyte polarity changes including microtubule destabilization, and increased monolayer permeability; in bile duct explants from neonatal pups, biliatresone treatment led to lumen obstruction and sub epithelial fibrosis^12–14^. Biliatresone is thus an *in vitro* and *ex vivo* tool that provides an unparalleled opportunity to glean new insight into the primary mechanism of BA.

Biliatresone binds strongly to reduced glutathione (GSH)^12,15^. It causes decreases in intracellular levels of GSH; artificial reductions in GSH through treatment with DL-buthionine sulfoximine (BSO) mimic the phenotype of biliatresone treatment. Interestingly, low GSH levels cause microtubule destabilization in other cell types^16–18^. Furthermore, in a recent study conducted on BA patients, oxidative damage positively correlated with BA incidence, liver inflammation, and cirrhosis^19^. We also found that biliatresone decreased mRNA levels of the transcription factor Sox17 and that Sox17 silencing phenocopied the effects of biliatresone^14^. The role of Sox17 in gallbladder and bile duct specification during embryogenesis is well known. Complete loss of Sox17 activity, which is embryonic lethal^20^, leads to failure of gallbladder formation and changes in the molecular phenotype of bile duct cells^21^. In Sox17 haploinsufficient mice, a BA-like phenotype with hypoplastic gallbladder epithelia detached cells from the luminal wall, leading to bile duct stenosis or atresia^22^. However, the relationship between Sox17 and GSH depletion and the details of the injury pathway downstream of biliatresone and GSH are still unknown.

In this work we identified three genes as key players in biliatresone-mediated cholangiocyte injury: RhoU/Wrch1, Hey2 and Sox17. We demonstrated that all are downstream of decreased GSH, and that RhoU/Wrch1 is upstream of Hey2, which in turn is upstream of Sox17. Our work provides a link between an external environmental insult and an intra-cellular signaling cascade involving key factors in cholangiocyte development and homeostasis. Understanding the cellular mechanisms underlying biliatresone-induced cholangiocyte injury, may provide valuable insight into BA in humans.

## Results

### **Biliatresone and decreased GSH alter RhoU/Wrch1, Hey2 and Sox17 expression**

In order to study the cellular mechanism of biliatresone-induced lumen obstruction, we first carried out a microarray analysis to compare biliatresone-treated primary neonatal mouse cholangiocytes to cells treated with vehicle or inactive precursors of biliatresone^23^. The microarray demonstrated biliatresone-specific changes in Notch signaling pathway genes including Hey2 (data have been deposited in NCBI’s Gene Expression Omnibus and are accessible through GEO Series accession number GSE136895). We also performed a Wnt signaling microarray (Qiagen) comparing cholangiocytes treated with biliatresone or vehicle (dimethyl sulfoxide; DMSO) and found that RhoU/Wrch1 was the most highly overexpressed.

RhoU/Wrch1 is a non-canonical Wnt signaling pathway member and atypical Rho GTPase. The Wnt pathway has been investigated in a wide range of liver pathologies and shown to regulate the repair of cholestatic liver injury^24,25^. It is a known regulator of cytoskeleton-related genes and actin organization. An increase in RhoU disrupts tight junction assembly in MDCK cells during epithelial cell polarization^26^. As biliatresone causes microtubule and tight junction changes, RhoU/Wrch1 appears to be an attractive candidate as a downstream mediator of the effect of biliatresone.

Hey2 is a Notch signaling effector protein. The Notch signaling pathway is known to be important in liver diseases and cholangiopathies^27–29^. It is critical for cell-cell communication and tissue homeostasis, via the regulation of a variety of cellular processes^30^.

**Figure 1:**
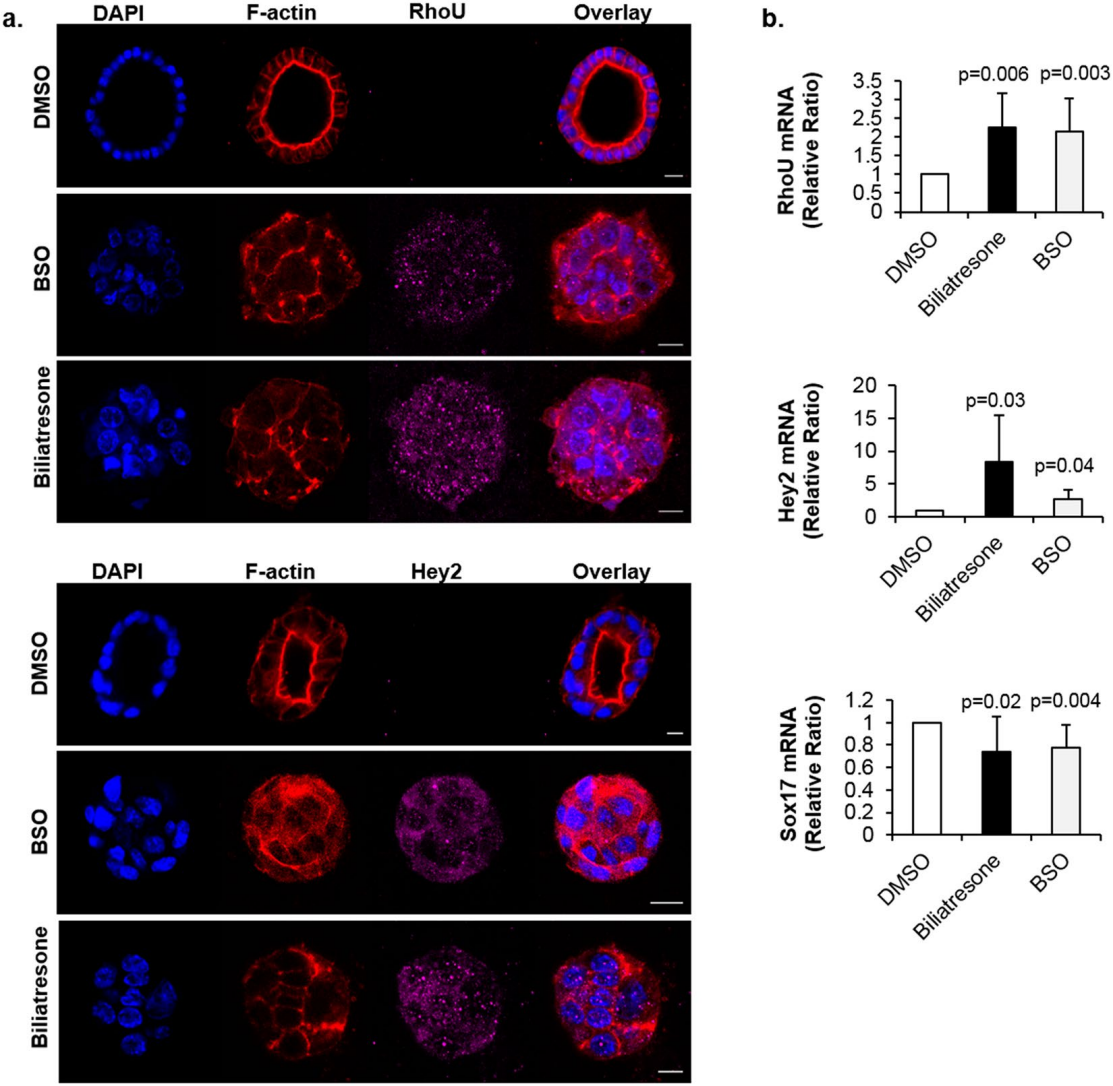
Biliatresone regulates RhoU/Wrch1, Hey2 and Sox17 expression in cholangiocytes. (**a**) Primary mouse neonatal extrahepatic cholangiocyte spheroids were treated with biliatresone (2 µg/ml), BSO (100 µM), or DMSO (same volume as biliatresone) for 24 h. Representative images of spheroids immunostained for F-actin (red), and RhoU/Wrch1 or Hey2 (magenta). Nuclei were stained with DAPI (blue). Images are representative of n = 4 independent experiments. Scale bar = 10 µm. (**b**) RhoU/Wrch1, Hey2 and Sox17 messenger RNA relative expression in cholangiocytes treated with biliatresone, BSO, or vehicle (DMSO) for 24 h (set as 1). Data represent mean ± s.e.m, n = 4-7 independent experiments; p values represent comparison to vehicle.

We thus further investigated the roles of Hey2 and RhoU/Wrch1 by treating mouse primary neonatal extrahepatic cholangiocytes in spheroid culture and a monolayer culture of small cholangiocyte cell line with biliatresone, BSO (which decreases cellular GSH), and DMSO (Figs. 1a, S1b). Both biliatresone and BSO treatment resulted in lumen obstruction in spheroid culture (as previously shown^14^) and caused increased expression of RhoU/Wrch1 and Hey2 at both mRNA and protein levels, as assessed by immunofluorescence staining, FACS analysis, and qRT-PCR. We confirmed these treatments also reduced Sox17 mRNA and protein expression (Figs. 1b, S1b).

**Figure 2:**
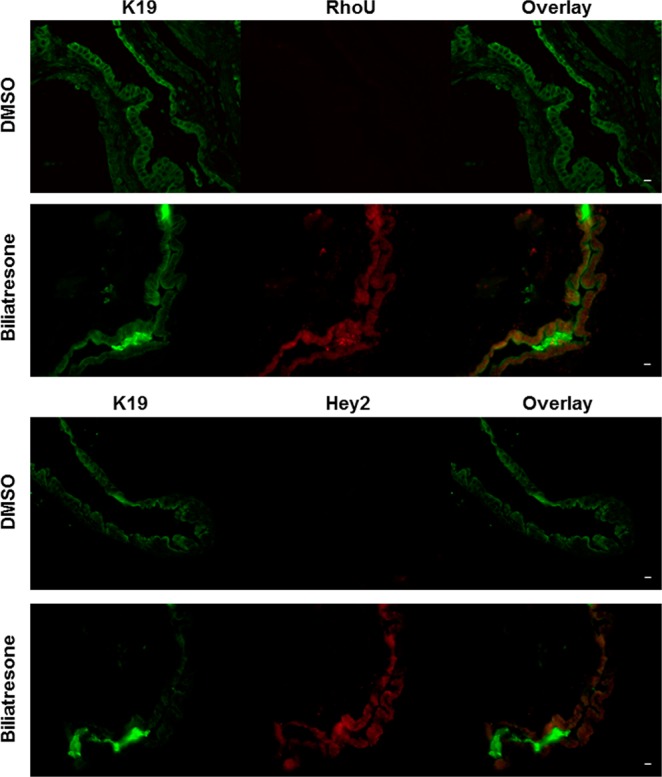
Biliatresone increases RhoU/Wrch1 and Hey2 expression in neonatal EHBD explants. Neonatal mouse bile duct explants were treated with DMSO (same volume as biliatresone) or biliatresone (2 µg/ml), for 24 h and stained for K19 (green), and RhoU/Wrch1 or Hey2 (both red). Scale bar = 10 µm, representative photos from n = 3 independent experiments.

In order to determine if the above-mentioned changes also occur in neonatal EHBDs, we dissected mouse neonatal EHBD explants and cultured them in a high oxygenation tissue incubator with biliatresone or vehicle for 24 h. EHBDs treated with biliatresone showed obstructed lumens with epithelial monolayer disruption and increased RhoU/Wrch1 and Hey2 expression compared to vehicle treatment (Fig. 2).

### RhoU/Wrch1 or Hey2 overexpression phenocopies the effects of biliatresone and BSO

**Figure 3:**
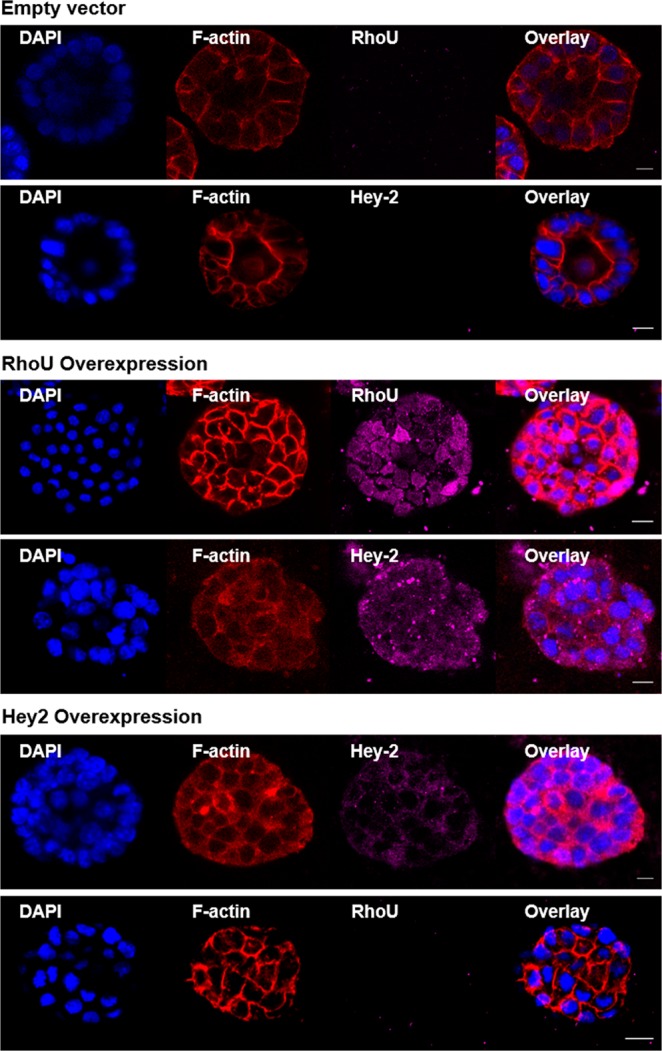
Overexpression of RhoU/Wrch1 and Hey2 in cholangiocyte spheroids mimics the effects of biliatresone and BSO. SBEC spheroids were transfected with RhoU/Wrch1- or Hey2-encoding plasmids. Cells were immunostained for F-actin (red), RhoU/Wrch1 or Hey2 (magenta), and DAPI (blue) 48 h after transfection. Images are representative of n = 5 independent experiments; scale bar = 10 µm.

We next determined whether changes in RhoU/Wrch1 and Hey2 are relevant to bile duct damage and lumen obstruction. We first overexpressed RhoU/Wrch1 and Hey2 using plasmid transfections of cholangiocytes in spheroids. Overexpression of both RhoU/Wrch1 and Hey2 resulted in lumen obstruction, and phenocopied the effects of biliatresone (Fig. 3). Moreover, overexpression of RhoU/Wrch1 also increased Hey2 expression, but not vice versa, suggesting that RhoU/Wrch1 is upstream of increases in Hey2. We previously showed that treatment with Sox17 siRNA also resulted in lumen obstruction and apical tight junction disruption^14^. Taken together, the data show that RhoU/Wrch1, Hey2, and Sox17 all play a role in cholangiocyte injury, where RhoU/Wrch1 is upstream of Hey2.

### Hey2 is upstream of Sox17 and RhoU/Wrch1 is upstream of both Hey 2 and Sox17

**Figure 4:**
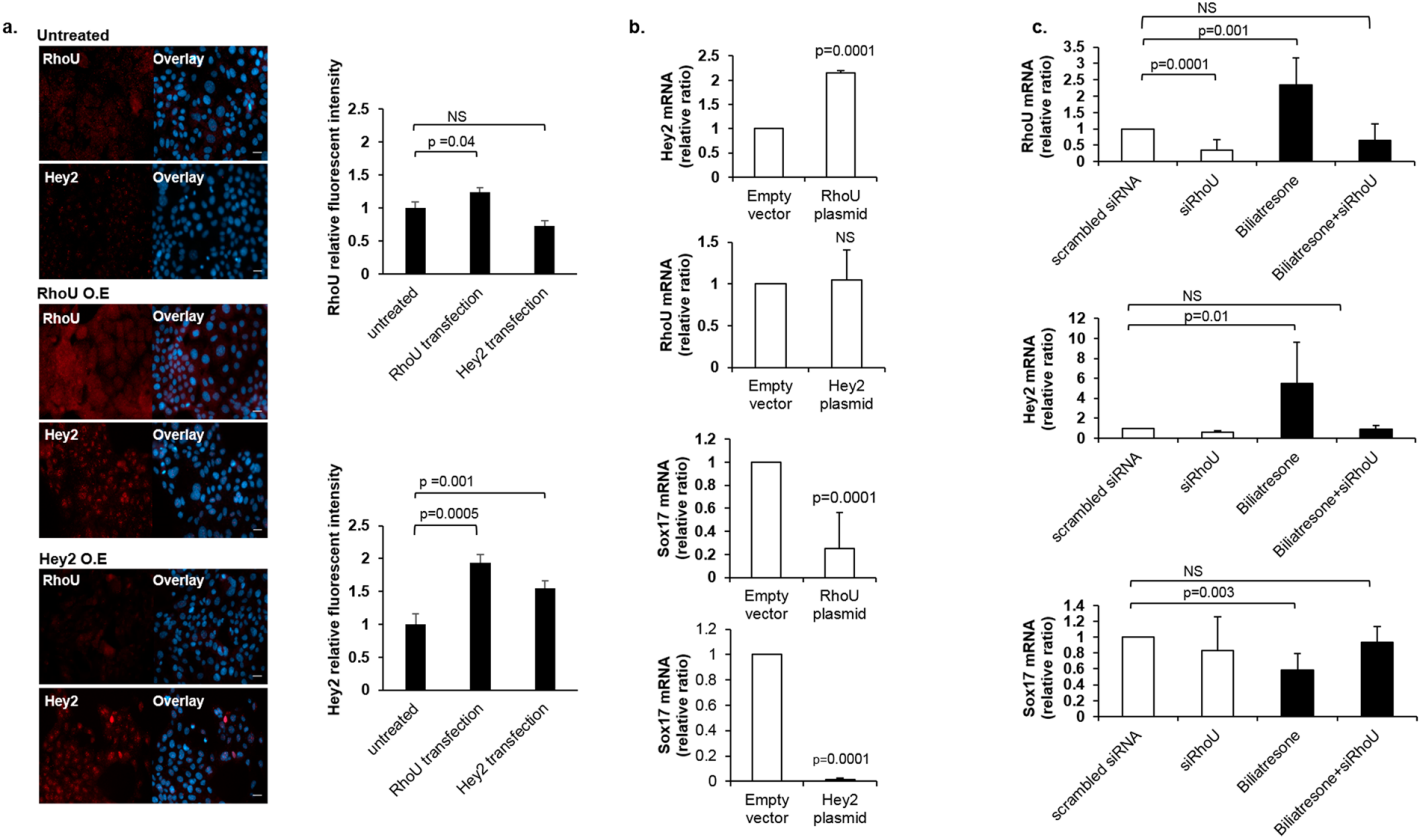
Hey2 is upstream of Sox17 and RhoU/Wrch1 is upstream of both Hey2 and Sox17. (**a**) SBEC cholangiocytes transfected with RhoU/Wrch1 or Hey2 plasmids and with an empty vector as a control. Cells were immunostained for RhoU/Wrch1 or Hey2 (red) 48 h after transfection. Nuclei were stained with DAPI (blue). Images are representative of n = 3 independent experiments. Relative fluorescence intensity was calculated from at least 20 fields for each condition. (**b**) SBECs transfected with RhoU/Wrch1 or Hey2 plasmids. RhoU/Wrch1 and Hey2 mRNA levels were evaluated by qRT-PCR 48 h after transfection. All graphs represent mean ± s.e.m, n = 3-7 independent experiments. NS = non-significant. (**c**) SBECs were treated with siRNA against RhoU/Wrch1 or with scrambled RNA for 48 h. After 24 h, cells were treated with biliatresone (2 µg/ml) or DMSO (same volume as biliatresone) for an additional 24 h. RhoU/Wrch1, Hey2, and Sox17 transcript levels were evaluated by qRT-PCR. Black histograms highlight biliatresone treatment; n = 4-10 independent experiments. All graphs represent mean ± s.e.m.

In order to further define the relationship between RhoU/Wrch1, Hey2, and Sox17, we compared RhoU/Wrch1 and Hey2 expression via immunofluorescent stains of cholangiocytes transfected with RhoU/Wrch1, Hey2, or empty plasmids. RhoU/Wrch1 overexpression resulted in elevated levels of Hey2 but not vice versa (similar to the results seen in spheroids; Fig. 4a). To verify the staining results, we conducted qRT-PCR analysis. Transfection with RhoU/Wrch1 plasmid increased Hey2 mRNA expression and decreased Sox17 mRNA expression (Fig. 4b). Of note, transfection with Hey2 did not affect levels of RhoU/Wrch1, but did result in decreased levels of Sox17 mRNA.

To determine whether biliatresone-mediated changes in Hey2 and Sox17 are downstream of RhoU/Wrch1, we used siRNA to prevent RhoU/Wrch1 upregulation in response to biliatresone (Fig. 4c). Silencing of RhoU/Wrch1 was effective, decreasing RhoU/Wrch1 mRNA levels by 70 percent (Fig. 4c). RhoU/Wrch1 mRNA levels increased by 230 percent following treatment with biliatresone (Fig. 4c); however, siRhoU/Wrch1 prevented the RhoU/Wrch1 biliatresone-induced increase, thus the level of RhoU/Wrch1 level was not significantly changed from baseline. Preventing biliatresone-induced increases in RhoU/Wrch1 abrogated changes in Hey2 and Sox17 (Fig. 4c). Taken together, these data imply that RhoU/Wrch1 is upstream of Hey2 and Sox17 in biliatresone-mediated pathways of injury, and Hey2 is upstream of Sox17.

### RhoU/Wrch1 and Hey2 are increased in IHBDs of BA patients

**Figure 5:**
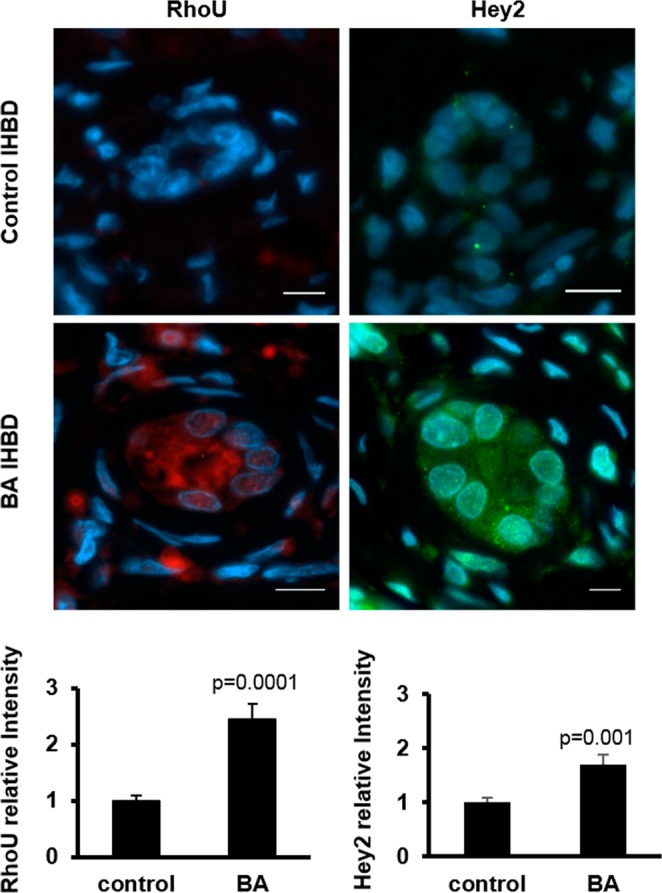
RhoU/Wrch1 and Hey2 are overexpressed in livers from BA patients. Liver biopsies from BA and non-BA patients (protocol biopsies from liver transplant patients) were immunostained with antibodies against RhoU/Wrch1 (red) or Hey2 (green). DAPI (blue) stain for nuclei. Graphs represent relative intensity taken from 50 images (total) in four biopsies of BA and three biopsies of non-BA patients respectively. scale bar = 10 µm.

To correlate our findings to human BA, we immunostained liver biopsies from BA and non-BA patients (four and three patients respectively) with antibodies against Hey2 and RhoU/Wrch1. Consistent with data from mouse cholangiocytes, there was an increase in RhoU/Wrch1 and Hey2 expression in cholangiocytes in biopsies from BA-affected compared to non-BA livers of both normal and Alagille patients (Figs. 5, S2).

## Discussion

**Figure 6:**
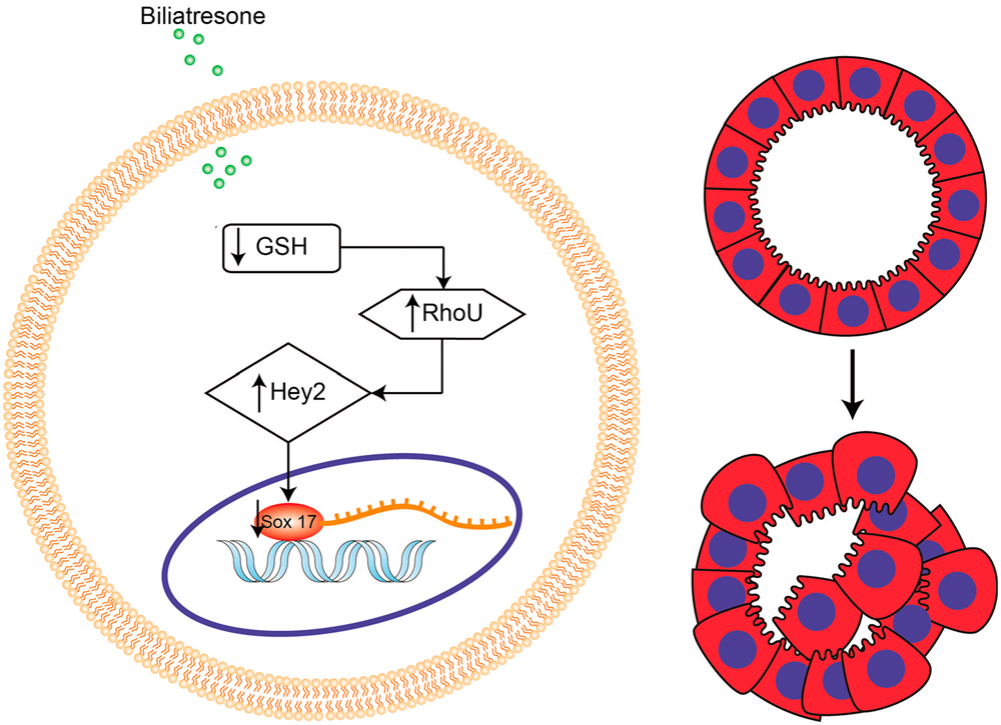
Model of proposed pathway of biliatresone-induced injury. Biliatresone causes decreased levels of cellular GSH which lead to upregulation of RhoU/Wrch1. This then increases Hey2 expression, which in turn causes decreased expression of the transcription factor Sox17, ultimately leading to cholangiocyte damage with loss of monolayer integrity.

We demonstrated that decreases in GSH initiated a pathway that resulted in increased RhoU/Wrch1, which in turn led to a sequential increase in Hey2 expression and decrease in Sox17 expression (Fig. 6). We used the biliary toxin biliatresone or the chemical BSO to decrease GSH in cells, and confirmed our findings through overexpression and knock down experiments and assays of both mRNA and protein expression. Our data overall suggest that GSH, RhoU/Wrch1, Hey2, and Sox17 comprise a linear signaling pathway that, when activated, leads to bile duct damage.

RhoU/Wrch1 is a member of the Wnt signaling pathway. RhoU/Wrch1 GTPases are transcriptionally induced by non-canonical Wnt signaling pathway and by and by gp130 cytokines through the JAK/STAT3 pathway^31^ and serve as regulators of cell organization and maintenance of the cytoskeleton. RhoU/Wrch1 plays an important role in epithelial architecture and morphogenesis in the foregut endoderm^32,33^ and in mouse mammary epithelial cells, in which overexpression of RhoU/Wrch1 results in morphological transformations^34^. In kidney epithelial cells, RhoU/Wrch1 expression is essential for the maintenance of cell morphogenesis; its upregulation has dramatic effects on the assembly of tight junctions^26^.

In our study, overexpression of RhoU/Wrch1 mimicked the effect of biliatresone on cholangiocyte spheroids and caused lumen obstruction. We previously showed that biliatresone treatment as well as decreases in GSH result in destabilization of cellular tubulin^14^. Microtubules are an essential part of the cytoskeleton, and the microtubule network is crucial for the maintenance of apical polarity^35^. Since RhoU/Wrch1 plays an important role in the regulation of cytoskeleton dynamics, this protein as well as other Wnt pathway members may link the effect of biliatresone to changes in the polarity of biliary epithelial cells.

Interestingly, in zebrafish embryos, glutathione peroxidase 4 acts as a suppressor of Wnt/β-catenin signals. It was shown that the depletion of glutathione peroxidase 4 (which decreases GSH) increases Wnt signaling^36^. This is consistent with our finding that biliatresone decreased GSH levels, which in turn led to increases in RhoU/Wrch1 expression.

Hey family proteins are basic, helix-loop-helix type transcription factors that mediate Notch signaling^37^. Hey proteins have an established role in intra-hepatic bile duct development and regeneration^38^. Here we showed that Hey2 is involved in bile duct injury and is downstream of RhoU/Wrch1, a Wnt signaling member. Sox17 is a transcription factor that regulates cholangiocyte differentiation and serves as a key protein in EHBD development and maintenance of the biliary tree^21,39^. The relationship between Sox17 and BA was first reported when Sox17 heterozygote mice developed BA-like disease^22^. We previously showed that biliatresone causes decreases in Sox17, and that knockdown of Sox17 in cholangiocyte spheroids mimics the effect of biliatresone^14^. Here we showed that Sox17 is downstream of decreased GSH and elevated RhoU and Hey2. Several other studies also suggest a link between Sox17 and Wnt signaling pathways. Interestingly, in different reports, it is reported as upstream or downstream of Wnt pathways. For example, inhibition of Sox17 in esophageal cancer activated Wnt Signaling^40^, and upregulation of Sox17 inhibited canonical Wnt signaling in gut endoderm^20,33,41^. On the other hand it was shown that β-catenin, a downstream Wnt signaling factor, can upregulate Sox17^42,43^.

In this study we identified three genes as key players in biliatresone-mediated cholangiocyte injury: RhoU/Wrch1, Hey2, and Sox17. We demonstrated that all are downstream of decreased GSH, and that RhoU/Wrch1 is upstream of Hey2, which in turn is upstream of Sox17. Our findings provide a link between an external environmental insult and an intra-cellular signaling cascade involving key factors in cholangiocyte development and homeostasis. Understanding the cellular mechanisms underlying biliatresone-induced cholangiocyte injury, may provide valuable insight into BA in humans. Further studies are needed to investigate whether this pathway is relevant to other cholangiopathies.

In summary, we identified several genes in a single pathway involved in cholangiocyte injury and bile duct obstruction. This pathway links redox stress to Wnt and Notch signaling pathway components and Sox17. These results suggest a novel molecular pathway of bile duct damage that may be amenable to previously unconsidered therapeutic interventions.

## Methods

### Use of experimental animals

All mice used in experiments were under a strict standard of care and experimental planning, covered by licensed approval from the Tel Aviv University institutional review board and the Israeli Ministry of Health (License number 01-16-098). BALB/c mice were obtained from ENVIGO. Both male and female mice were used for all analyses.

### Human samples

Anonymized human liver biopsy slides were obtained from the Anatomic Pathology archives of the Children’s Hospital of Philadelphia (Pennsylvania, USA), with IRB approval. Tissue Samples of Biliary Atresia patients were obtained at time of diagnosis. Control biopsies were biopsies read as normal by the pathologist.

### Cell culture

Primary neonatal (3 days old) extrahepatic cholangiocytes or a small intrahepatic cholangiocyte cell line (SBEC) were used for 2D or 3D spheroid culture as previously described^14,44^. Cells were cultured with biliary epithelial cell (BEC) medium^45^ and incubated at 37 °C with 5% CO_2_.

### Spheroid culture

Primary extrahepatic cholangiocytes and SBEC were cultured in 3D in a collagen-Matrigel mixture as described previously^12,14,23^. Cholangiocytes in 3D culture replicate, polarize, and form hollow spheroids with apical markers on the luminal side and basolateral markers on the external side after 7–8 days. Spheroids were used for experiments at day 8 after plating.

### Neonatal bile duct explant culture

Intact EHBDs were isolated from 0- to 3-day-old or from 3-week-old BALB/c mice according to a protocol modified from that described previously^45^.

Biliatresone or vehicle were added to the media, and ducts were placed on roller inserts and cultured in a Vitron Dynamic Organ Culture Incubator at 37 °C, in 95% O_2_ and 5% CO_2_, for 24 h. Bile ducts were then embedded in a solution of 2% bactoagar and 2.5% gelatin, allowed to solidify at 4 °C for 1 h, then transferred to histology cassettes containing 70% alcohol. Gels were processed to paraffin blocks and sectioned for staining (see below, fluorescent staining).

### Transfection

Transfections of SBEC, primary cholangiocytes, or spheroid cultures were performed with Lipofectamin 2000 for plasmids (Invitrogen, 11668019) and Lipofectamin RNAiMAX for siRNA (Invitrogen, 13778-030) following the manufacturer’s instructions. Plasmids were: RhoU/Wrch1 (GeneCopoeia, EX-W0152-M02), Hey2 (GeneCopoeia, EX-Mm03016-M45), or empty vector (GeneCopoeia pRecieiver-M45 expression clone). Cells transfected with an empty plasmid with the same backbone served as controls for transfection experiments. For siRNA experiments, the RhoU/Wrch1 Dharmacon ON-Targetplus siRNA (CAAGCAAACUUCCGAGAAC) was used. Cells transfected with non-targeting siRNA served as controls for these experiments.

### Biliatresone treatments

Biliatresone was synthesized as described^46^. Cells were treated with vehicle (DMSO) or biliatresone (2 µg/ml) or BSO (100 µM, Sigma, 19176) for 24 h unless otherwise noted. This concentration was chosen after based on our previous study. We tested 5 different dosages ranging from 0.125 µg/ml to 2 µg/ml, and measured the percentage of affected spheroids out of all spheroids and found the 2 µg/ml is most effective in causing lumen obstruction^23^.

### Microarray

Primary neonatal extrahepatic cholangiocytes were used for experiments after 6–8 days of growth, and the cells were equally split into the respective wells (totaling 10^4^ cells/ well). 48 h after plating, the cholangiocytes were treated with vehicle control, and biliatresone, at a dose of 2 µg/ml for 6 hours. Five independent experiments were performed. After completion of the incubation, the cells were removed with 0.25% Trypsin, washed once, flash frozen with liquid nitrogen, and stored in −80 °C freezer for RNA isolation at a later time. On the day of isolation, cells were thawed on ice, and RNA isolation was performed immediately with the RNA Micro Kit (Qiagen 74004). The RNA was submitted to University of Pennsylvania Microarray Core, where further analysis was done using the Affymetrix 1-cycle Mouse Gene 1.0ST. K19 staining was done (1:10, Developmental Studies Hybridoma Bank, University of Iowa), as a measure of purity of each of the submitted samples.

### Immunofluorescence staining

#### Spheroid culture

Treated spheroids were fixed with 4% paraformaldehyde (PFA), blocked with permeabilization solution (10% FBS, 0.5% Triton X-100 in PBS), and stained for various fluorescent markers: F-actin (1:1000; phalloidin tetramethyl rhodamine B isothiocyanate; Santa Cruz Biotechnology, 301530) and RhoU/Wrch1 (Abcam, ab80315) or Hey2 (Proteintech, 10597-1-AP). Spheroids were cultured with primary antibodies diluted with permeabilization solution overnight in 4 °C, washed with permeabilization solution, incubated with secondary antibodies for 3 h in RT, washed 5 times, fixed again with PFA for 30 min in 37 °C, stained with DAPI (5 min, RT), then washed and placed on a slide with mounting medium.

Images were obtained using a Leica SP5 confocal microscope with 40X magnification. Images were taken at the level of the midsection of each spheroid, where the luminal diameter was greatest. Inserts had between 20 and 80 spheroids for all experiments, and a minimum of five images was taken for each condition, with representative images shown in the figures.

#### Cholangiocyte 2D culture

SBECs (10^5^ cells in 500 µl per well in 24 well plates) were grown in 8-well chamber slides (LAB-TEK II Nunc, Thermo Fisher Scientific). Cells were treated with biliatresone, BSO or DMSO for 24 h and fixed with 4% PFA (30 min 37 °C). In transfection experiments with plasmids for overexpression, cells were fixed after 48 h in the same manner. Cells were then washed three times with PBS, permeabilized by incubation in 0.1% Triton X100 for 4 min at RT, and blocked for 1 h at RT in PBT buffer (2% BSA, 0.2% TritonX100, and 2% goat serum in PBS). Cells were stained with primary antibodies diluted in PBT (45 min at RT), washed and stained with secondary antibody diluted in PBT (30 min at RT), stained with DAPI (D1306 Invitrogen) (1:1000, 5 min), and mounted on slides using Fluorescent Mounting Medium (KPL 71-00-16). Cells were visualised using an Axioimager Z2 apotome microscope, at 40×. Images were analysed using ImageJ software, Win64 version: https://imagej.net/Fiji/Downloads.

#### Staining of paraffin embedded sections

Slides with paraffin-embedded tissue sections were deparaffinized by warming to 60 °C, treated with xylene, then rehydrated with decreasing concentrations of ethanol (100%, 95%, 80%, 70%). Slides were incubated in microwave oven for 15 min in citric acid buffer (pH 6), cooled, and washed in running water, washed with PBS, blocked with PBT, and cultured with primary antibodies diluted in PBT overnight at 4 °C, washed with PBT, incubated with secondary antibodies for 30 min in RT, washed two times, stained with DAPI (5 min, RT), washed, and covered with mounting medium and a cover slip. Images were taken using a Leica SP5 confocal microscope at 40X magnification.

### Flow cytometry analysis

SBECs (2 * 10^5^ cells in 2 ml per well in six well plates) were plated overnight at 37 °C to allow cell adhesion. On the following day, cells were treated with biliatresone or BSO for 24 h. Staining for the intracellular proteins RhoU/Wrch1 (1:300), Hey2 (1:100), and Sox17 (1:100, Abcam, ab192453) was done in eBioscience Foxp3/Transcription Factor Staining Buffer Set (Invitrogen, 00-5523-00) for 1 h at RT. This intracellular staining was followed by PE-conjugated goat anti-mouse or PE-conjugated donkey anti-rabbit for 1 h at RT. Cells were then centrifuged and resuspended in FACS buffer (PBS, 5% FBS, 0.1% azide). The stained cells were examined using a Gallios Flow cytometer, and analysed using Kaluza Analysis 2.1 Software (Beckman Coulter) https://www.beckman.com/flow-cytometry/software/kaluza

### Real-time quantitative PCR (qRT-PCR)

SBECs (3*10^5^ cells in 2 ml per well in six well plates) were plated overnight at 37 °C to allow cell adhesion. On the following day, cells were treated with biliatresone, BSO, or DMSO for 24 h. For overexpression experiments, cells were transfected with RhoU/Wrch1, Hey2 plasmids, or empty plasmid. For silencing experiments, cells were treated with RhoU/Wrch1 small interfering RNA (ON-TARGET plus Mouse RhoU/Wrch1 siRNA (Dharmacon, LQ-064428-01-0002 NM_133955) or scrambled siRNA (ON-TARGETplus Non-targeting Pool, Dharmacon, D-001810-10-05), as per manufacturer’s instructions for 48 h. Total RNA from SBECs was extracted with the EZ-10 DNAaway RNA Mini-Preps Kit (Bio Basic) according to the manufacturer’s protocol. cDNA was prepared using a qScript cDNA Synthesis kit (Quantabio). Probe sets including *RhoU/Wrch1 (Mm00505976_m1), Hey2 (Mm01180513_m1), Sox17 (Mm00488363_m1) and GAPDH* endogenous control (Mm99999915_g1), were TaqMan pre-developed assay reagents from ABI. Real-time quantitative PCR was performed using the StepOnePlus Real-time PCR system.

### Antibodies

Additional antibodies used for cholangiocyte characterization and signal transduction genes included: keratin 19 (K19, 1:10, Developmental Studies Hybridoma Bank, TROMAIII), collagen I (1:200; Southern Biotech, 1310-01), α-smooth muscle actin (mouse α-SMA, Abcam, ab7817). Secondary antibodies: PE (anti-mouse eBioscience, 12-4010-82, anti-rabbit #12-473981), APC (anti-mouse eBioscience, 17-4010-82). Alexa secondary antibodies- Abcam: goat anti-rabbit 647 (ab150083), goat anti-rat 568 (ab175710), goat anti-rabbit 555 (ab150086).

### Statistical analysis

Values are presented as mean± s.e.m unless otherwise indicated; a two-tailed student t-test was used for comparison. A significant difference between groups was defined as *p* < 0.05. All experiments were conducted at least 3 times with duplicates (number specified at each experiment).

All methods were carried out in accordance with relevant guidelines and regulations.

## Supplementary information

Supplementary Information.
