# Supplementary material for: Extrahepatic cholangiocyte obstruction is mediated by decreased glutathione, Wnt and Notch signaling pathways in a toxic model of biliary atresia
Source: Sci Rep. 2020 May 5;10:7599. doi: 10.1038/s41598-020-64503-5 (PMC7200694; doi:10.1038/s41598-020-64503-5)
Supplement: Supplementary file 1 — Supplementary Information. [file 41598_2020_64503_MOESM1_ESM.pdf]

Supplementary Information

Extrahepatic cholangiocyte obstruction is mediated by GSH, Wnt and Notch signaling pathways in a toxic model of biliary atresia

Sophia Fried[1,2], Daphna Gilboa[1,2], Adi Har-Zahav[1,2], Yu Du[3], Sara Karjoo[4], Pierre Russo[5], Raanan Shamir[1,2], Rebecca Wells[3], Orith Waisbourd-Zinman[1,2,6]

[1] Institute for Gastroenterology, Nutrition and Liver Diseases, Schneider Children's Medical Center of Israel, Petach Tikva, Israel.

[2] Sackler Faculty of Medicine, Tel-Aviv University, Tel-Aviv, Israel.

[3] Department of Pathology, Hospices Civils de Lyon, Lyon, France.

[4] Division of Gastroenterology, Department of Medicine, Perelman School of Medicine at the University of Pennsylvania, Philadelphia, PA, United States.

[5] Johns Hopkins School of Medicine, Baltimore, Maryland, United States.

[6] Department of Pathology and Laboratory Medicine, The Children's Hospital of Philadelphia, Philadelphia, PA, United States.

[7] Division of Gastroenterology, Hepatology, and Nutrition, Department of Pediatrics, The Children's Hospital of Philadelphia, Philadelphia, PA, United States.

Correspondence to: Orith Waisbourd-Zinman, MD

14 Kaplan st. Petach Tiqva, Israel 49202

Tel: +972-432077679; Fax: +972-39253104; E-mail: oritwz@gmail.com

Fig. S1

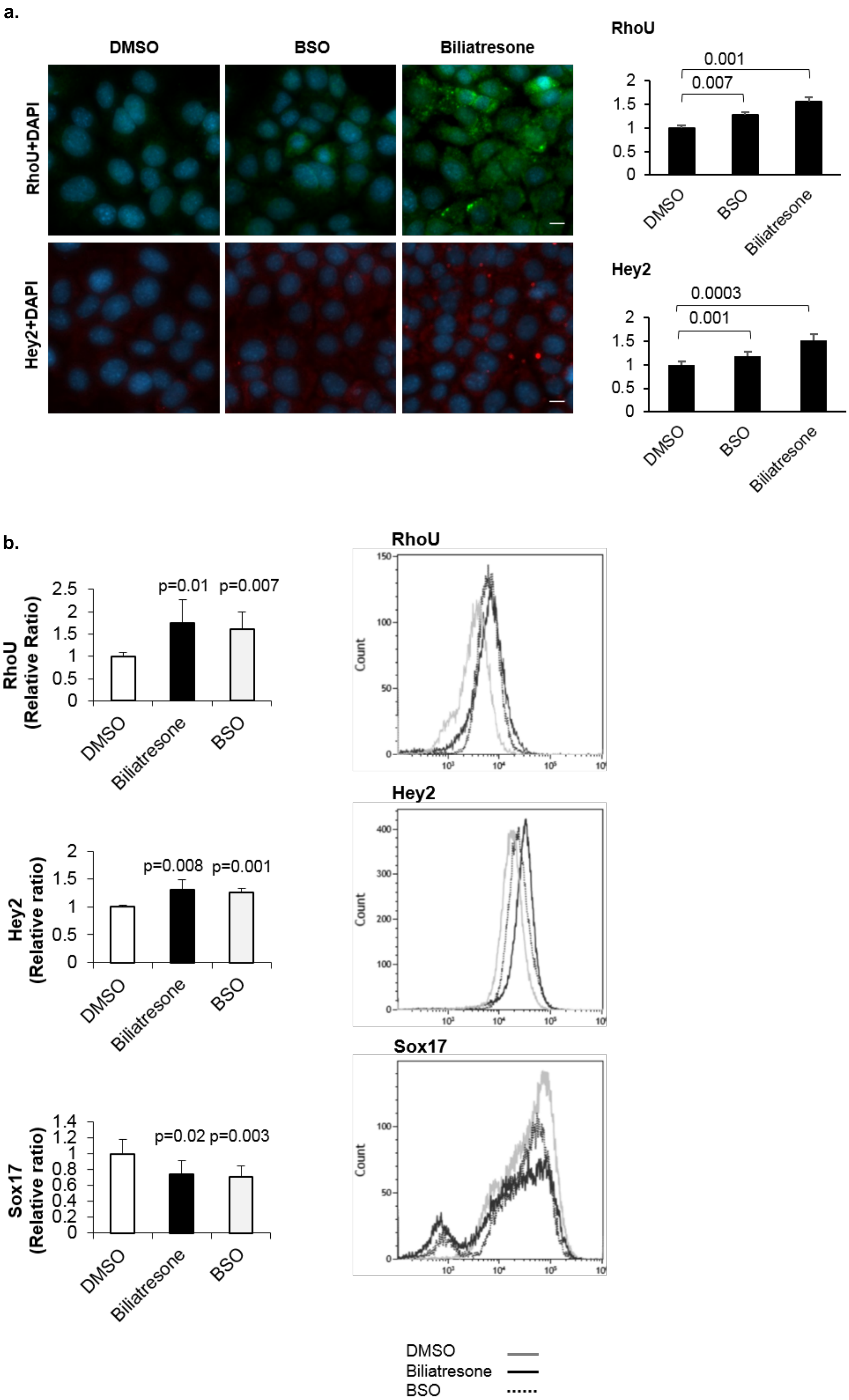
a.
DMSO
BSO
Biliatresone
RhoU+DAPI
Hey2+DAPI
RhoU
0.001
0.007
2
1.5
1
0.5
0
DMSO
BSO
Biliatresone
Hey2
0.0003
0.001
2
1.5
1
0.5
0
DMSO
BSO
Biliatresone
b.
RhoU (Relative Ratio)
2.5
2
1.5
1
0.5
0
p=0.01 p=0.007
DMSO
Biliatresone
BSO
Hey2 (Relative ratio)
2
1.5
1
0.5
0
p=0.008 p=0.001
DMSO
Biliatresone
BSO
Sox17 (Relative ratio)
1.4
1.2
1
0.8
0.6
0.4
0.2
0
p=0.02 p=0.003
DMSO
Biliatresone
BSO
RhoU
Count
Hey2
Count
Sox17
Count
DMSO
Biliatresone
BSO

Fig. S1. Biliatresone regulates RhoU and Hey2 expression in cholangiocyte culture

a. sBEC cells were cultured in the presence or absence of 2ug/ml Biliatresone or 100uM BSO for 3h. Left: RhoU and Hey2 protein levels were evaluated by immunofluorescence. DAPI (blue), RhoU (green), Hey2 (red) Right: quantitative analysis of relative Fluorescent intensity. The images are representative of 4 experiments and values of MFI were calculated from at least 40 fields of cells for each experimental group. Bars represent mean ± s.e.m, Bar = 10 µm.

b. RhoU/Wrch1, Hey2, and Sox17 protein levels in cholangiocytes treated with biliatresone, BSO or DMSO, as evaluated by flow cytometry. Histogram graph on the left represents mean fluorescent intensity relative to DMSO (set as 1), graphs on the right represent histograms showing the cell count (Y axis) that show a given fluorescence, and the relative fluorescence intensity detected in each condition (X-axis). Data represent mean ± s.e.m, n=5-9 independent experiments; p values represent comparison to vehicle.

Fig. S2

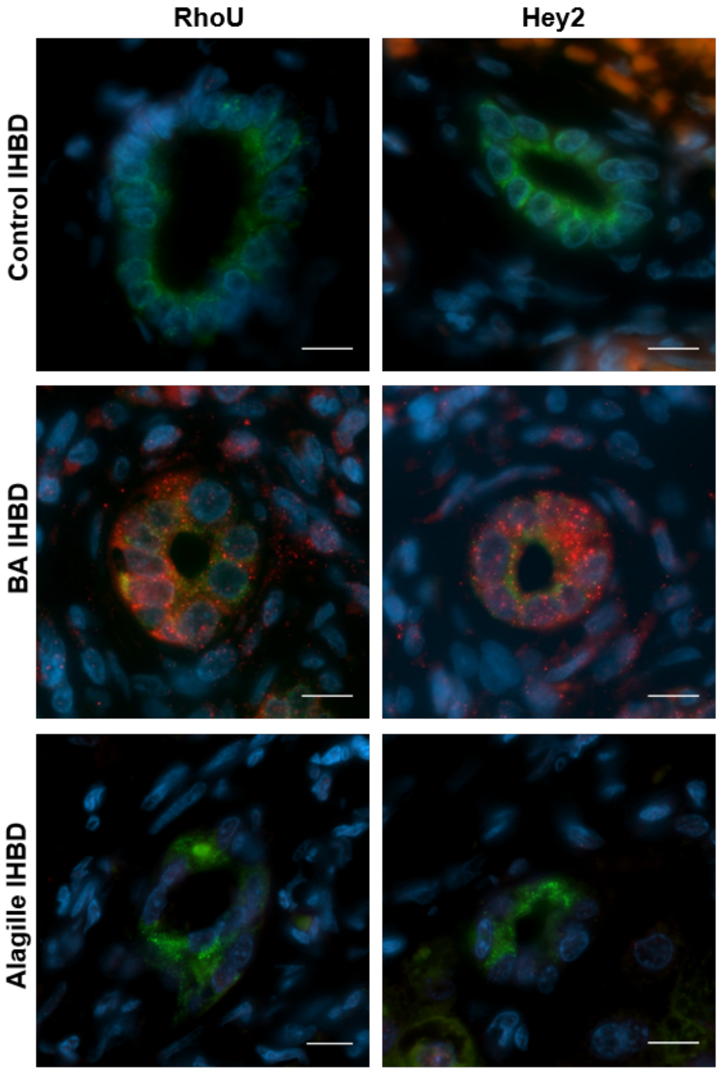

Fig. S2. RhoU/Wrch1 and Hey2 are overexpressed specifically in Intra hepatic bile ducts in liver biopsies from BA patients but not in Alagille patients or normal biopsies.

Liver biopsies from non-BA patients, BA patients and Alagille patients, were immunostained with antibodies against K19 (green) RhoU/Wrch1 (red) and DAPI (blue) stain for nuclei. The images are representative of 3 experiments. Bar = 10 μm.
